# Personality traits in psychosis and psychosis risk linked to TSPO expression: a neuroimmune marker

**DOI:** 10.1017/pen.2020.14

**Published:** 2020-11-24

**Authors:** Cory Gerritsen, Yajur Iyengar, Tania DaSilva, Alex Koppel, Pablo Rusjan, R. Michael Bagby, Romina Mizrahi

**Affiliations:** 1 Forensic Early Intervention Service, Centre for Addiction and Mental Health, Toronto, Ontario, Canada; 2 Research Imaging Centre, Centre for Addiction and Mental Health, Toronto, Ontario, Canada; 3 School of Interdisciplinary Science, McMaster University, Hamilton, Ontario, Canada; 4 Douglas Research Centre, McGill University, Montreal, Quebec, Canada; 5 Department of Psychology, University of Toronto, Ontario, Canada

**Keywords:** Positron Emission Tomography, TSPO, Neuroinflammation, Personality, Psychosis

## Abstract

Personality has been correlated with differences in cytokine expression, an indicator of peripheral inflammation; however, the associations between personality and central markers of inflammation have never been investigated *in vivo* in humans. Microglia are the resident macrophages of the central nervous system, and the first responders to tissue damage and brain insult. Microglial activation is associated with elevated expression of translocator protein 18kDa (TSPO), which can be imaged with positron emission tomography (PET) to quantify immune activation in the human brain. This study aimed to investigate the association between personality and TSPO expression across the psychosis spectrum. A total of 61 high-resolution [^18^F]FEPPA PET scans were conducted in 28 individuals at clinical high risk (CHR) for psychosis, 19 First-Episode Psychosis (FEP), and 14 healthy volunteers (HVs), and analyzed using a two-tissue compartment model and plasma input function to obtain a total volume of distribution (V_T_) as an index of brain TSPO expression (controlling for the rs6971 TSPO polymorphism). Personality was assessed using the Revised NEO Personality Inventory (NEO-PI-R). We found TSPO expression to be specifically associated with neuroticism. A positive association between TSPO expression and neuroticism was found in HVs, in contrast to a nonsignificant, negative association in CHR and significant negative association in FEP. The TSPO-associated neuroticism trait indicates an unexplored connection between neuroimmune activation and personality that varies across the psychosis spectrum.

Abnormal immune response is suggested to contribute to the underlying pathology of schizophrenia. Supporting this, genome-wide association studies have reported associations between immune-related genes and schizophrenia; in particular, genetic markers observed across the major histocompatibility complex have been reliably associated with genetic risk for schizophrenia (Schizophrenia Working Group of the Psychiatric Genomics Consortium, 2014; Stefansson et al., 2009). In addition, peripheral markers of inflammation including cytokines have been shown to be elevated in schizophrenia (Goldsmith, Rapaport, & Miller, 2016). Studies of individuals with clinical high risk (CHR) for developing schizophrenia have similarly reported elevated levels of pro-inflammatory markers such as cytokines and C-reactive proteins (CRPs) (Perkins et al., 2014; Stojanovic et al., 2014).

Immune response in the brain can be studied *in vivo*, using positron emission tomography (PET) and radiotracers that target 18 kDa translocator protein (TSPO), a protein that is over-expressed by glia during brain immune response (Venneti, Wiley, & Kofler, 2009) and may play a role in hormonal and behavioral stress responses according to recent research using mouse models (Fukudome et al., 2018; Zhang et al., 2018). Studies using the first-generation radiotracer [^11^C] PK11195 suggested higher TSPO expression in schizophrenia (Doorduin et al., 2009; van Berckel et al., 2008) or equivalent binding relative to controls (Di Biase et al., 2017; Holmes et al., 2016; van der Doef et al., 2016). However, the use of both [^11^C] PK11195 and binding potential as an outcome measure has been criticized (e.g., Plaven-Sigray et al., 2018). Studies using newer tracers have shown predominantly null results (Bloomfield et al., 2016; Coughlin et al., 2016; Hafizi et al., 2016; Kenk et al., 2015; Ottoy et al., 2018; Takano et al., 2010) with one study (Collste et al., 2017) showing reduced TSPO in individuals with schizophrenia-spectrum diagnoses. A meta-analysis incorporating individual-level data (i.e., a “mega-analysis”) was conducted by Plaven-Sigray et al. (2018) including only studies using the second-generation tracers and the validated total distribution volume (V_T_) as the outcome measure. This analysis sought to resolve some of the methodological issues of prior work with improved power, revealing significantly lower TSPO across the brain of individuals with early psychosis. While contrary to hypotheses of increased neuroinflammation in psychosis, these findings support an as-yet-unknown role for differential neuroinflammatory function in the highly heterogeneous psychosis-spectrum population.

In CHR samples, two studies have been conducted using second-generation radiotracers and V_T_ as outcomes (Bloomfield et al., 2016; Hafizi et al., 2017). Neither of these revealed differences between CHR and healthy volunteers (HVs).

Beyond its putative role in psychosis, inflammation is also associated with the additional psychological phenotype of personality. Currently, the Five-Factor Model (FFM; Costa & MacCrae, 1992) is the most well-explored and validated model of personality in psychiatric literature, with extensive support from genetics, psychopathology, and other areas of research (Widiger & Crego, 2019). It has demonstrated applicability to psychiatric samples (Bagby et al., 1999). The inflammatory marker interleukin-6 (IL-6) has shown a complex relationship with two personality traits – conscientiousness and neuroticism – within the FFM. Sutin et al. (2010) found a positive association between IL-6 and neuroticism, and a negative association with conscientiousness; no associations were found for the remaining three traits – extraversion, openness, and agreeableness. These authors reported similar associations between FFM traits and CRP, another inflammatory marker. Turiano, Mroczek, Moynihan, and Chapman (2013) produced somewhat different results, showing a similar negative relationship between IL-6 and conscientiousness but only in the context of high neuroticism (i.e., IL-6 was decreased only in individuals with both high conscientiousness *and* high neuroticism). Chapman et al. (2009) further found a negative association between IL-6 and extraversion, while Chapman et al. (2011) and Luchetti, Barkley, Stephan, Terracciano, and Sutin (2014) found negative associations between IL-6 and the traits of conscientiousness and openness. Potential implications of these findings for schizophrenia-spectrum diagnoses arise from the observation that schizophrenia is consistently associated with high neuroticism and low scores on the remaining FFM traits (openness, agreeableness, conscientiousness, and extraversion; Ohi et al., 2016). To date, no study has investigated personality and brain neuroimmune function.

These observations suggest the possibility of interactions between personality and neuroinflammatory markers in the schizophrenia spectrum. From a clinical standpoint, the clarification of these relationships may inform biomarker development, and hold promise to improve prognostic work; especially when examined across the full spectrum of illness progression. In this study, for the first time, we aim to investigate whether personality traits are linked to TSPO expression across the spectrum of HVs, psychosis risk, and psychosis. Based on findings reviewed above from peripheral inflammation markers showing inflammation–personality associations in nonpsychiatric populations, we hypothesize a positive association between TSPO expression and neuroticism and a negative association between TSPO expression and extraversion, openness, and conscientiousness. Given the evidence for higher levels of neuroticism in the schizophrenia spectrum and the previously established link between peripheral inflammatory markers in those with high neuroticism, lower central neuroinflammation in those with schizophrenia-spectrum psychopathology relative to controls is counterintuitive, and the relationship between neuroinflammation and neuroticism across the spectrum is therefore hypothesized to vary. This is the first time the relationship between personality traits and central neuroinflammation has been examined, and the exact nature of this predicted variance cannot yet be specified.

## 1. Methods

### 1.1 Participants

A total of 61 PET scans in 28 CHR participants, 19 First-Episode Psychosis (FEP) participants (6 antipsychotic naïve), and 14 HVs were included in this study. All participants have been reported in previous TSPO PET studies (Hafizi et al., 2016, 2017), however, the correlation between TSPO and personality traits is unique to the present study and has not been previously reported. Demographic and clinical characteristics can be found in Table 1.

**Table 1.** Participant study population characteristics and [^18^F]FEPPA injection parameters

		HV (*n* = 14)	CHR (*n* = 28)	FEP (*n* = 19)	
Age (SD)		21.5 (1.6)	21.2 (3.3)	26.9 (6.7)	*F* = 10.54, *p* < .001
Gender	Female	10	13	5	*χ*^2^ = 6.612, *p* = .037
	Male	4	15	14	
Genotype	HAB	10	19	15	*χ*^2^ = 0.697, *p* = .706
	MAB	4	9	4	
PET measures	Amount inj. (SD) (mCi)	4.98 (0.35)	5.06 (0.26)	5.14 (0.24)	*F* = 1.541, *p* = .223
	Specific Activity (SD) (mCi/µmol)	1933.52 (2099.13)	1923.96 (2094.631)	2862.20 (2795.21)	*F* = 1.048, *p* = .357
	Mass inj. (SD) (µg)	1.88 (1.47)	1.83 (1.53)	1.04 (0.53)	*F* = 2.584, *p* = .0841

Eligibility of CHR, FEP, and HV participants in this study followed the inclusion criteria described in previous studies (Hafizi et al., 2016, 2017). In short, CHR individuals had to fulfill the diagnostic criteria as described by the Criteria of Prodromal Syndromes established using the Structured Interview for Prodromal Symptoms (Miller et al., 2002). To be included, FEP patients must have been diagnosed with schizophreniform disorder, delusional disorder, schizophrenia, or psychotic disorder not otherwise specified, using the SCID-IV (First, Spitzer, Gibbon, & Williams, 1997) with minimal to no antipsychotic exposure. HVs with any history of psychiatric illness or first-degree relatives with a major mental disorder were excluded. Participants were additionally excluded if they presented with any of the following: pregnancy or currently breastfeeding; a clinically significant medical illness; or the presence of metal implants that would preclude an MRI scan.

This study was approved by the Research Ethics Board at the Centre for Addiction and Mental Health (CAMH). The authors assert that all procedures contributing to this work comply with the ethical standards of the relevant national and institutional committees on human experimentation and with the Helsinki Declaration of 1975, as revised in 2008.

### 1.2 Personality measurement

**Figure 1.**
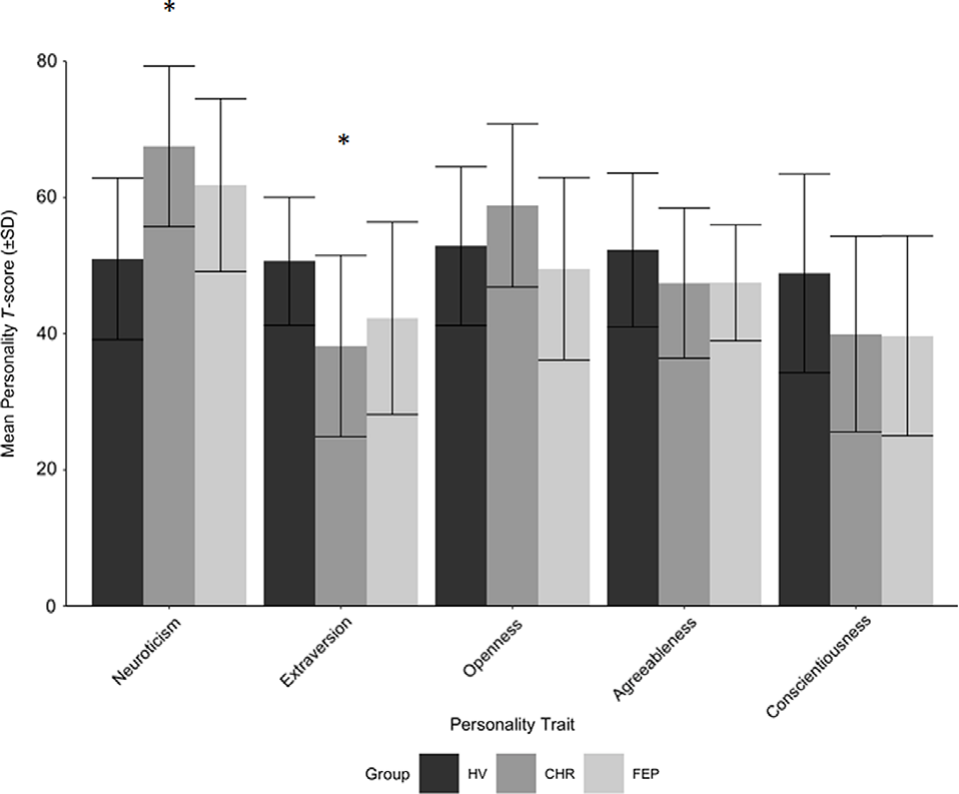
Participant study population mean personality scores, as scored using the NEO-PI-R. (*) indicates significance after correction for family-wise error using the Hochberg procedure.

Participant personality traits were measured using the Revised NEO Personality Inventory (NEO-PI-R; Costa & MacCrae, 1992), the mostly used measure of universal personality traits in the psychiatric literature (Widiger & Crego, 2019). This is a 240-item questionnaire in which personality traits are grouped into five factors, or dimensional domains, in accordance with the FFM. Responses to the items of the NEO-PI-R are made on a five-point Likert-style scale, ranging from “strongly disagree” to “strongly agree.” The five domains include Neuroticism, Extraversion, Agreeableness, Openness, and Conscientiousness. Following methods of Costa & MacCrae (1992), the raw scores collected from the questionnaire were transformed into *T*-scores adjusted for age and sex differences using the published normative pool data. Sample mean personality scores can be found in Figure 1.

### 1.3 Image processing and calculation of total distribution volumes (V_T_) following PET image acquisition and analysis

PET and MRI data acquisition have been described in detail elsewhere (Hafizi et al., 2016, 2017). Briefly, each participant underwent a [^18^F]FEPPA PET scan for 125 min on a high-resolution CPS-HRRT PET scanner, and a proton density (PD)-weighted brain MRI scan using a 3T MR-750 scanner (General Electric Medical Systems, Chicago, IL, USA). Arterial blood samples were collected automatically for the first 22.5 min at a rate of 2.5 mL/min after radioligand injection using an automatic blood sampling system (model PBS-101, Veenstra Instruments, Joure, the Netherlands). Manual blood samples were also taken at −5, 2.5, 7, 12, 15, 20, 30, 45, 60, 90, and 120 min relative to the time of injection to measure radioactivity in blood, and determine the relative proportion of radiolabeled metabolites. The dispersion-and metabolite-corrected plasma input function was generated as previously described (Mizrahi et al., 2012). Time–activity curves were extracted for the cerebellum, dorsolateral prefrontal cortex (DLPFC), and medial prefrontal cortex (MPFC) using a validated in-house imaging pipeline (Rusjan et al., 2006). These ROIs were chosen based on previous literature reporting relevant alterations in TSPO and on their established importance to schizophrenia-spectrum disorders (Da Silva, Hafizi, Rusjan et al., 2019; Da Silva, Hafizi, Watts et al., 2019; Hafizi et al., 2018). All regions of interest were delineated using a proton density MRI in each participant. [^18^F]FEPPA distribution volume (V_T_) was derived from the time–activity curve using a two-tissue compartment model and plasma input function, which has been validated for [^18^F]FEPPA quantification (Rusjan et al., 2011). All participants were grouped based on their TSPO rs6971 polymorphism as high-(C/C), mixed-(C/T), or low-affinity (T/T) binders (HAB, MAB, and LAB, respectively), as previously described (Owen et al., 2012).

### 1.4 Statistical analyses

All analyses were performed using the Statistical Package for the Social Sciences (SPSS) Version 24.0 (Armonk, NY: IBM Corp.). Demographic measures were compared across groups using chi-square tests for categorical variables and analysis of variances (ANOVAs) for continuous variables. Five one-way ANOVAs were used to determine whether there were main effects of group on any NEO trait *T*-score. Then, a series of three multivariate ANOVAs (MANOVAs) including continuous (TSPO V_T_, age) and categorical (group, rs6971 polymorphism, and sex) independent variables were used to predict each NEO trait, which were entered as dependent variables. These tests were conducted via the SPSS General Linear Model (GLM) suite. This allowed testing of any main effects of TSPO V_T_ on each NEO trait, and allowed any differences in the association between NEO *T-*scores and TSPO V_T_ between groups to be examined by testing group × TSPO V_T_ interactions (no other interaction terms were included in the models to conserve degrees of freedom). The rs6971 polymorphism was added to the models in order to control for its known effect on TSPO ligand-binding affinity (Mizrahi et al., 2012; Owen et al., 2012). Sex and age were included to control for their potential effects as well. An alpha level of .05 was chosen. Significant main effects for group, and significant interaction effects between group and TSPO V_T_, in the three MANOVAs were followed by examining univariate ANOVA results within-trait. Significant findings in each case were corrected for family-wise error using the Hochberg method.

## 2. Results

### 2.1 Demographics and injection parameters

A summary of the demographic and clinical characteristics of each group is presented in Table 1. Participant age was unevenly distributed among groups (*F*_2,58_ = 10.54*, p* < .001), as was participant sex (*X*^2^_2,61_ = 6.612, *p* = .037). There were no significant differences between groups with regard to PET radiotracer injection parameters or TSPO genotype (all *p > .05*).

### 2.2 Differences in personality, and association between [^18^F]FEPPA VT and personality, between CHR, FEP, and HVs

Main effects of the group on each NEO trait were significant only for neuroticism (*F*_2,58_ = 8.685, *p* = .001) and extraversion (*F*_2,58_ = 4.413, *p* = .02) (Table 2, Figure 1). The main effect of group on openness did not survive Hochberg correction (*p* = .04, α = .017), and those for agreeableness and conscientiousness were nonsignificant (*p* = .3 and .1, respectively).

**Table 2.** Summary of mean personality trait *T*-scores for study samples

	HV (*n* = 14)	CHR (*n* = 28)	FEP (*n* = 19)	
Neuroticism (SD)	51 (11.86)	67.5 (11.8)	61.79 (12.69)	*F* = 8.685, *p* < .01^*^
Extraversion (SD)	50.64 (9.39)	38.18 (13.31)	42.26 (14.13)	*F* = 4.413, *p* = .02^*^
Openness (SD)	52.86 (11.65)	58.82 (11.99)	49.53 (13.39)	*F* = 3.367, *p* = .04
Agreeableness (SD)	52.29 (11.3)	47.43 (11.03)	47.47 (8.51)	*F* = 1.172, *p* = .32
Conscientiousness (SD)	48.86 (14.59)	39.93 (14.36)	39.68 (14.67)	*F* = 2.089, *p* = .13

* Indicates significance after correction for family-wise error using the Hochberg procedure.

Main effects for [^18^F]FEPPA V_T_ on NEO traits were not significant in any model. Testing of the interaction between group and [^18^F]FEPPA V_T_ effects on the five NEO traits revealed that the association between [^18^F]FEPPA V_T_ and NEO traits was significantly different between groups within the DLPFC (*F*_10,98_ = 2.135, Pillai’s Trace = .358, *p* = .028, α = .05). In the cerebellum, the effect did not survive Hochberg correction (*F*_10,98_ = 1.957, Pillai’s Trace = .333, *p* = .046, α = .025). In the MPFC, the effect did not reach significance (*F*_10,98_ = 1.836, Pillai’s Trace = .316, *p* = 0.064, α = .017).

To follow-up on the significant group × [^18^F]FEPPA V_T_ interaction in the DLPFC model, univariate effects for each NEO trait within this model were examined. Testing of the interaction between group and [^18^F]FEPPA V_T_ effects on neuroticism revealed a significant interaction, (*F*_2,52_ = 5.126, *p* = .009, α = .05). All other univariate interaction effects in this model were nonsignificant. While the omnibus MANOVA for the cerebellum model did not survive Hochberg correction, the univariate effect on neuroticism in this model was examined for comparison and was similar in magnitude and direction (*F*_2,52_ = 4.919, *p* = .011).

**Figure 2.**
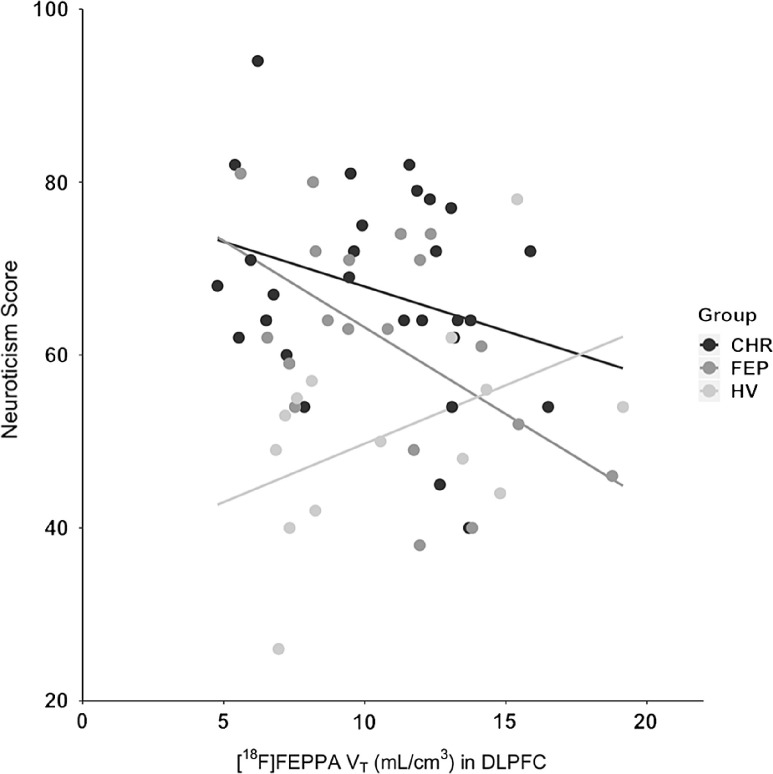
Associations between [^18^F]-FEPPA TSPO binding (V_T_) and neuroticism in the DLPFC.

Post hoc testing was conducted to explore the significant interaction between group and [^18^F]FEPPA V_T_ effects on neuroticism in DLPFC. The model was broken down into three pairwise models; one for each pair of clinical groups, maintaining control for the effects of rs6971 polymorphism, age, and sex. Interaction terms were then examined for each pairwise contrast and corrected for family-wise error using the Hochberg procedure. HV individuals showed a positive association between [^18^F]FEPPA V_T_ and neuroticism, in contrast to a negative association seen in FEP and CHR (Figure 2). The association between [^18^F]FEPPA V_T_ and neuroticism was significantly different when comparing HVs and CHR individuals (*F*_1,26_ = 9.690, *p* = .004, α = .05) and when comparing HVs and FEP individuals (*F*_1,26_ = 9.132, *p* = .006, α = .025), but not when comparing FEP and CHR individuals (*F*_1,26_ = 0.886, *p* = .352).

## 3. Discussion

The primary aim of this study was to investigate whether personality traits are linked to TSPO expression, a neuroimmune marker, in the spectrum from health to psychosis and psychosis risk. The hypothesis of a positive association between neuroticism and TSPO expression was supported in the HV group, but not in the CHR or FEP groups, who in fact showed null or significantly negative associations. The results currently observed in HV individuals are consistent with existing literature showing an association between elevated neuroticism and increases in peripheral inflammation in other nonpsychiatric samples (e.g., Sutin et al., 2010).

The hypothesis of negative associations between TSPO expression and other FFM personality traits were not supported, as all other relationships were nonsignificant. The final hypothesis of variability in the association between TSPO expression and neuroticism across the spectrum was supported by a compelling crossover interaction driven by the reversal of associations between these two variables from HV (positive associations) to CHR and FEP samples (negative associations). This interaction effect was similar across ROIs but was only significant for the DLPFC after correction for family-wise error. While it is tempting to infer a specific role for the DLPFC in processes underlying this effect, then, evidence does not support specificity and a generalized effect may be observed across ROIs in a more powerful study.

These results may help account for the counterintuitive observation that peripheral markers of inflammation tend to be elevated among those high in neuroticism, while a recent meta-analysis revealed lower central neuroinflammation among those with schizophrenia-spectrum diagnoses (Plaven-Sigray et al., 2018), who in turn tend to show elevated neuroticism (Ohi et al., 2016). From a spectrum point of view, the results represent a continuum of varying relationships between central neuroinflammation from health, through illness proneness, to the full expression of psychotic disorder. The nature of this continuous variability is unknown given the cross-sectional nature of the present data, and may represent trait-like differences among individuals with varying degrees of disorder proneness or severity, or an ontogenetic progression as the disorder develops across the lifespan. Future longitudinal research may be useful to evaluate this hypothesis. A parallel relationship between TSPO expression and another potential biomarker for psychosis, the mismatch negativity event-related potential, has been observed by Banati and Hickie (2009); these authors found variability in this relationship between healthy controls and participants with schizophrenia, and thus described the potential utility of TSPO as a nondiagnostic marker of continuous variability between health and illness.

The pathological mechanism behind this variability across the schizophrenia spectrum is also unclear and requires future research. Other accounts for the lack of consistency in associations between peripheral and central markers of inflammation must also be considered. The suggestion by Coughlin et al. (2016) of increases in markers of inflammation within the CSF of recently diagnosed schizophrenia patients in the absence of altered TSPO expression, for example, illustrates the potential independence of these markers.

While the current findings require replication, and are primarily of interest due to their implications for basic psychopathology, they may hold downstream clinical implications. For example, expanded knowledge of the relationship between personality and neuroinflammation may be used in biomarker development, especially given the clinical utility of combining biomarkers from diverse sources in predicting onset and progression in psychosis (e.g., Schmidt et al., 2017).

Despite consistency between the current results and those of previous literature, several limitations inherent to PET studies need to be considered. First, the sample size for this study was small for associations among multiple variables. The nature of this study introduced a large number of exploratory correlations that may reduce the statistical power of the final associations following correction procedures. The exploration of facet-level interactions that may drive the relationship observed between neuroticism and neuroinflammation may be permitted by the collection of larger samples in future studies. However, this study is the largest PET study to date investigating brain neuroimmune function *in vivo* in the psychosis spectrum. Replication with larger samples and methodological improvements may address these issues, although such research may be limited due to the high cost of PET.

Second, though increases in [^18^F]FEPPA V_T_ are associated with microglial activation, other studies have observed additional factors associated with TSPO expression; for example, perivascular cells, vascular endothelial cells, and astrocytes express TSPO (Banati, Myers, & Kreutzberg, 1997; Notter et al., 2017), as do brain regions with substantial stem cell turnover (Betlazar, Harrison-Brown, Middleton, Banati, & Liu, 2018). Further, brain tissue remodeling in response to peripheral injury is associated with increased TSPO expression (Banati, 2002; Banati et al., 2001), suggesting that TSPO expression may not necessarily be correlated with other central markers for inflammation such as altered brain inflammatory cytokine expression. Further, microglial activation can occur in the absence of TSPO (Banati et al., 2014). While [^18^F]FEPPA is among the best-validated and most specific TSPO radioligands currently available, some reviews have called attention to these and other potential limitations (see Banati et al., 2014; Cumming et al., 2018; Liu et al., 2014).

In conclusion, we observed significant differences in the association between neuroticism and TSPO V_T_ when comparing HVs with those on the schizophrenia spectrum. These suggest a reversed relationship between neuroticism and neuroinflammation as assessed via TSPO expression between groups at differing points on this spectrum, with HVs showing the expected positive relationship and those in CHR and FEP groups showing the opposite. These findings may help clarify contradictory observations of low TSPO expression in the schizophrenia spectrum in spite of elevated neuroticism. These findings also support the importance of examining such pathophysiological findings across illness spectra, given the progressive differences in association detected currently.
